# Heart Failure and Cognitive Impairment Through the Lens of the Gut Microbiome: A Narrative Review

**DOI:** 10.3390/jpm15120595

**Published:** 2025-12-03

**Authors:** Ali Reza Rahmani, Seyed Avid Madani, Ethan Aminov, Lasha Gogokhia, Travis Bench, Andreas Kalogeropoulos

**Affiliations:** 1 Division of Cardiology, Department of Medicine, Stony Brook University, Stony Brook, NY 11790, USA; alireza.rahmani@stonybrookmedicine.edu (A.R.R.);; 2 Tehran Heart Center, Cardiovascular Diseases Research Institute, Tehran University of Medical Sciences, Tehran 1411713138, Iran; 3 Renaissance School of Medicine, Stony Brook University, Stony Brook, NY 11790, USA; 4 Division of Gastroenterology and Hepatology, Department of Medicine, Stony Brook University, Stony Brook, NY 11790, USA; 5 Health Sciences Center, Stony Brook University Medical Center, Stony Brook, NY 11794, USA

**Keywords:** heart failure, cognitive impairment, gut microbiome, Alzheimer’s disease, TMAO, SCFA, gut–heart–brain axis

## Abstract

Heart failure (HF) affects over 55 million individuals globally, with prevalence projected to exceed 11 million in the United States by 2050 and is increasingly recognized as a systemic disorder extending beyond hemodynamic dysfunction to encompass profound alterations in neural and gut physiology. Cognitive impairment affects nearly half of HF patients and represents a major determinant of morbidity, self-care capacity, and mortality. Recent advances suggest that the gut microbiome serves as a pivotal intermediary in the heart–brain crosstalk, influencing neurocognitive outcomes through inflammatory, metabolic, and neurohumoral pathways. Dysbiosis in HF disrupts intestinal barrier integrity, facilitating translocation of endotoxins and microbial metabolites such as trimethylamine-N-oxide (TMAO), short-chain fatty acids (SCFAs), and bile acids, which in turn modulate neuroinflammation, cerebral perfusion, and neuronal signaling. The gut–heart–brain axis provides an integrative framework linking HF and cognitive impairment pathophysiology through dysbiosis-driven systemic inflammation and metabolite dysregulation. Gut-derived biomarkers and microbiome-targeted interventions represent promising strategies for detection of early alterations and precision treatment, highlighting the urge for prospective, multi-omics studies to establish causality and therapeutic efficacy. This review synthesizes current evidence connecting gut microbiome dysbiosis and metabolite alterations to both HF and cognitive impairment pathophysiology and proposes translational strategies for integrating microbiome-targeted therapies in HF patients with cognitive dysfunction.

## 1. Introduction

Heart failure (HF) is a growing public health problem, with global prevalence more than doubling from approximately 25.4 million in 1990 to 55.5 million in 2021 [1]. In the United States, an estimated 6.7 million adults currently live with HF, with projections exceeding 11 million by 2050 [2]. HF remains one of the leading causes of hospitalization among adults over 65 years of age, with the lifetime risk of developing HF approaching 1 in 4 Americans [2,3]. The economic burden is substantial, with total HF-related direct medical expenditures in the United States expected to increase from $32 billion in 2020 to $142 billion by 2050, driven largely by hospitalizations [2]. These data underscore the escalating burden of HF and the need for earlier detection, precision prevention, and disease-modifying interventions to alter its trajectory.

Beyond its cardiovascular consequences, HF imposes a substantial neurocognitive burden that extends its impact far beyond the heart. Cognitive impairment is now recognized as a major comorbidity in HF, contributing to reduced functional independence, poorer self-care, and higher rates of hospitalization and mortality [4,5,6,7,8]. The prevalence of cognitive impairment among patients with HF is highly variable, ranging from 20% to 80%, depending on the study population, HF phenotype, and cognitive assessment tools used [4,5,9]. Population-based studies indicate that patients with HF have a two to threefold higher risk of developing cognitive decline or dementia compared with age-matched controls without HF [4,10,11]. In a recent multicenter study of younger and middle-aged patients with acute HF (under age 65), cognitive impairment was observed in 19.6% of participants and was associated with significantly higher 30-day risk of cardiovascular death or rehospitalization (HR 1.52, 95% CI 1.07–2.17) [12]. In a meta-analysis of ~3 million individuals across 29 studies, Vishwanath et al. reported that HF was associated with poorer performance across global cognition, memory, executive function, attention, and language domains (Hedges’ g ranging from –0.50 to –0.73), and conferred an 80% increased risk of cognitive impairment (HR 1.80, 95% CI 1.14–2.86) [13]. The burden is particularly pronounced among older adults, those with reduced cardiac output, and patients with multiple comorbidities such as diabetes and chronic kidney disease [9]. These findings emphasize that cognitive impairment is an important and clinically relevant consequence of HF, warranting its integration into comprehensive management strategies and individualized risk assessment.

While the cardiovascular and neurocognitive sequelae of HF are well recognized, recent evidence suggests that both may be influenced by the gut, which serves as a central hub in systemic health and disease [14,15]. The gut–heart and gut–brain axes have been independently studied, each revealing critical insights into the bidirectional communication between the gastrointestinal tract and these vital organs. In HF, the gut–heart axis has been implicated in disease progression through mechanisms involving reduced intestinal perfusion, venous congestion, and gut barrier dysfunction, which promote translocation of microbial metabolites such as trimethylamine N-oxide (TMAO) and lipopolysaccharides (LPS), potent mediators of inflammation and myocardial remodeling [16,17,18,19]. Separately, the gut–brain axis has been shown to modulate neurocognitive health via immune, endocrine, and neural signaling pathways, where gut dysbiosis and metabolite imbalance contribute to neuroinflammation, microglial activation, and cognitive decline [20,21,22]. Although previous reviews have explored the gut–heart or gut–brain axes independently, few have addressed their intersection. This review is distinctive in integrating both axes into a unified framework, emphasizing how gut microbiome may simultaneously influence cardiovascular and cognitive function in HF. Together, these independent lines of evidence have converged to suggest that the gut may represent a common mechanistic link between cardiac dysfunction and cognitive impairment, forming the foundation for the emerging concept of the gut–heart–brain axis.

## 2. Methods

A narrative review methodology was used to synthesize the existing literature on the topic. A comprehensive search of PubMed/MEDLINE, Embase, and Google Scholar was conducted. Search terms included combinations of Medical Subject Headings (MeSH) and free-text keywords such as: “heart failure”, “cognitive impairment”, “dementia”, “Alzheimer’s disease”, “gut microbiome”, “microbiota”, “gut–heart–brain axis”, “TMAO”, “trimethylamine N-oxide”, “SCFA”, “short-chain fatty acids”, “inflammation”, “neuroinflammation”. Boolean operators (“AND”, “OR”) were applied to optimize search sensitivity and specificity. The literature search was performed independently by A.R.R and S.A.M. The search covered studies published from database inception to October 2025. The findings were evaluated using a quality appraisal approach, and the most relevant studies were selected. Studies were excluded if they were non-English, conference abstracts, duplicate publications, or papers unrelated to the core themes of the gut–heart–brain axis, microbial metabolites, heart failure, or cognitive impairment. Titles and abstracts of all retrieved articles were initially screened, after which the full texts of potentially eligible papers were obtained and thoroughly reviewed by authors.

## 3. The Gut–Heart Axis

**Figure 1:**
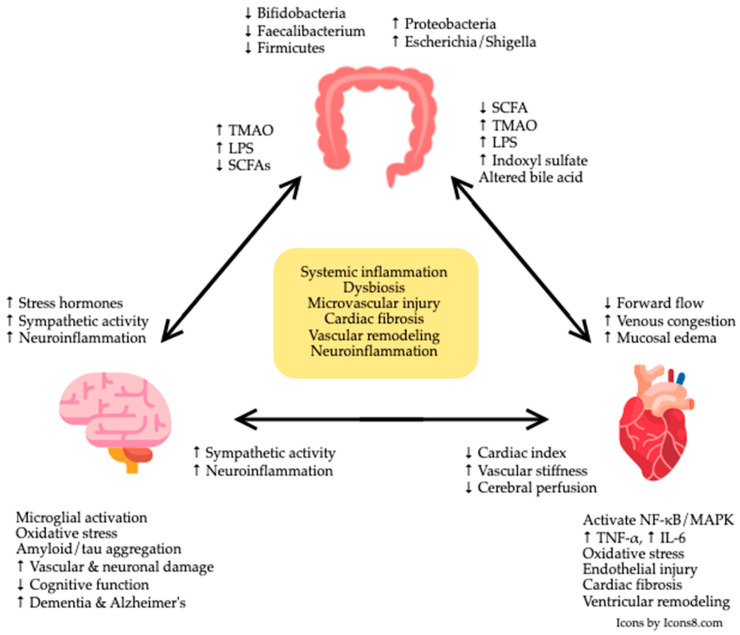
Interplay Between Gut Dysbiosis, Cardiac Dysfunction, and Cognitive Decline. TMAO, trimethylamine N-oxide; LPS, lipopolysaccharides; SCFAs, short-chain fatty acids; NF-κB, nuclear factor kappa-light-chain-enhancer of activated B cells; MAPK, mitogen-activated protein kinase; TNF-α, tumor necrosis factor-alpha; IL-6, interleukin-6.

In HF, the interplay between cardiac dysfunction and gastrointestinal physiology, often termed the gut–heart axis, has appeared as a critical aspect of disease progression [16]. Hemodynamic abnormalities inherent to HF, particularly reduced cardiac output and elevated venous pressures, create a hostile environment in the intestine [23]. Diminished forward perfusion deprives the intestinal mucosa of adequate oxygen and nutrients, while venous congestion stimulates bowel wall edema and raises hydrostatic pressure in the splanchnic circulation [24]. Together, these insults compromise the integrity of the intestinal barrier, leading to increased gut permeability and local inflammation. Once the intestinal barrier is compromised, microbial products normally confined to the gut lumen can translocate into the systemic circulation [25]. Bacterial endotoxin (LPS) and other pathogen-associated molecules entering the circulation from the gut engage innate immune receptors, toll-like receptor 4 (TLR4), on immune cells, thereby triggering systemic inflammatory cascades and the release of pro-inflammatory cytokines [26]. Consequently, HF is often accompanied by chronic low-grade systemic inflammation; circulating levels of inflammatory mediators such as tumor necrosis factor-α and interleukin-6 are elevated and tend to correlate with disease severity [27]. Systemic immune activation driven by gut-derived endotoxins may depress myocardial function and accelerate remodeling [28]. This bidirectional interplay creates a vicious cycle wherein hemodynamic stress from HF fuels intestinal inflammation, which in turn worsens cardiac performance (Figure 1).

### 3.1. Microbiomes

The gut microbiome, a dense and metabolically active ecosystem comprising trillions of microorganisms, plays a pivotal role in maintaining systemic homeostasis [29]. In healthy individuals, commensal bacteria regulate nutrient absorption, energy metabolism, and immune balance through the production of short-chain fatty acids (SCFAs), bile acid metabolites, and other bioactive compounds that influence vascular tone and inflammatory signaling [29,30]. This finely tuned microbial community also maintains intestinal barrier integrity and prevents systemic translocation of pro-inflammatory molecules [30].

In the context of cardiovascular health, the gut microbiome contributes to the regulation of lipid metabolism, glucose homeostasis, and endothelial function, processes that influence vascular integrity and cardiometabolic risk [31]. Patients with HF exhibit a distinct gut microbiota profile characterized by disrupted microbial balance relative to healthy individuals [32]. However, in HF, this symbiotic relationship becomes disrupted. Hemodynamic alterations, including intestinal hypoperfusion and venous congestion, create a hostile luminal environment characterized by mucosal edema, ischemia, and altered oxygen gradients [16,33]. These changes foster microbial dysbiosis, typically marked by a reduction in beneficial taxa such as Faecalibacterium prausnitzii and Ruminococcaceae, and an expansion of pro-inflammatory or pathogenic species such as Enterobacteriaceae and Streptococcaceae [16,33,34]. The composition and diversity of the gut microbiota are increasingly recognized as important factors influencing systemic and cardiovascular health. In healthy individuals, the intestinal ecosystem is dominated by beneficial commensal bacteria such as Lactobacillus, Bifidobacterium, Faecalibacterium prausnitzii, and members of the Ruminococcaceae and Lachnospiraceae families, which together maintain metabolic, immune, and vascular equilibrium [33]. These microorganisms contribute to host physiology through the fermentation of dietary fibers into SCFAs, which reinforce epithelial barrier integrity, regulate blood pressure via endothelial nitric oxide signaling, and suppress pro-inflammatory cytokine production [16,33]. In a study, patients with HF with preserved ejection fraction (HFpEF) exhibited significant alterations in gut microbiota composition, including reduced α-diversity and depletion of SCFAs, producing taxa such as Ruminococcus, independent of age, body mass index, hypertension, and dietary fiber intake [35]. In another study in HF with reduced ejection fraction (HFrEF) patients, Katsimichas et al. [36] reported that individuals with nonischemic HFrEF display a distinct pattern of gut microbial imbalance, featuring an overrepresentation of *Streptococcus* and *Veillonella*, and notable alterations in microbial gene pathways linked to amino acid, carbohydrate, and vitamin. Pasini et al. [37] demonstrated that patients with chronic HF exhibit marked alterations in gut flora, characterized by an overgrowth of pathogenic species such as Campylobacter, Shigella, Salmonella, Yersinia enterocolitica, and Candida, alongside a reduction in beneficial commensals like Faecalibacterium prausnitzii and Blautia. These microbial shifts correlate with increased intestinal permeability and systemic inflammation, highlighting a transition toward a pro-inflammatory gut environment that may exacerbate HF progression.

In a recent study [34], patients with HF exhibit a significant reduction in alpha diversity, accompanied by depletion of Firmicutes and expansion of Proteobacteria, Actinobacteria, and Synergistetes phyla that harbor numerous opportunistic and pro-inflammatory species. At the genus level, HF cohorts display enrichment of Enterococcus, Streptococcus, Veillonella, Escherichia, and Klebsiella, all of which can produce endotoxins and reactive oxygen species, thereby amplifying systemic inflammation [34]. Conversely, populations of SCFA-producing commensal microbes such as Faecalibacterium prausnitzii, Eubacterium rectale, Dorea longicatena, and members of the Ruminococcaceae and Lachnospiraceae families, are markedly reduced [34]. As part of growing evidence linking gut dysbiosis to HF, Wang et al. [38] demonstrated that elderly patients with chronic HF exhibited reduced gut microbial diversity and distinct serum metabolomic shifts. In a seminal study, Kummen et al. [39] identified species-level shifts in HF, including increased Escherichia coli, Klebsiella pneumoniae, Streptococcus spp., and Enterococcus faecalis, alongside reduced Faecalibacterium prausnitzii, Eubacterium rectale, Ruminococcus bromii, and Bacteroides fragilis, reflecting a transition toward a pathobiont-rich microbiota that promotes barrier dysfunction and systemic inflammation. A recent systematic review [40] identified consistent alterations in gut microbial composition among patients with HF, including enrichment of pro-inflammatory taxa such as Ruminococcus gnavus and Escherichia/Shigella, alongside depletion of butyrate-producing genera like Faecalibacterium and Prevotella; these shifts were associated with elevated levels of LPS, TMAO, and indoxyl sulfate, implicating gut dysbiosis in systemic inflammation and disease progression. Moreover, using Mendelian randomization, Dai et al. identified a causal association between the gut microbial species Bacteroides dorei and HF, with apolipoprotein B mediating approximately 10.1% of this effect [41]. Taken together, these findings underscore the central role of gut microbiome dysbiosis in HF, highlighting its contribution to systemic inflammation, barrier disruption, and cardiometabolic deterioration through species-level shifts and loss of symbiotic microbial functions (Table 1).

### 3.2. Metabolites

The gut microbiota functions as a metabolic organ, generating bioactive molecules that profoundly influence cardiovascular physiology. In HF the profile of these microbial metabolites becomes pathologically skewed toward pro-inflammatory and cardiotoxic compounds [42].

Among the most studied is TMAO, produced via hepatic oxidation of microbial-derived trimethylamine from dietary choline, carnitine, and phosphatidylcholine [16,43]. Elevated plasma TMAO concentrations correlate with adverse outcomes, including myocardial fibrosis, diastolic dysfunction, and increased cardiovascular mortality. TMAO activates several pro-inflammatory signaling cascades that serve as common pathways in both cardiac and neurological pathology. Specifically, TMAO engagement leads to activation of nuclear factor-κB (NF-κB), mitogen-activated protein kinase (MAPK), and NLRP3 inflammasome pathways, which promote endothelial injury, increase production of pro-inflammatory cytokines (TNF-α, IL-6, IL-1β), and drive maladaptive remodeling [16,17,18,33,44]. Elevated plasma TMAO levels have been shown to independently predict long-term mortality in patients with stable HF, even after adjusting for B-type Natriuretic Peptide (BNP) and renal function [45], whereas Suzuki et al. [46] reported that in acute HF, higher circulating TMAO concentrations were significantly associated with in-hospital and one-year mortality, enhancing prognostic discrimination beyond traditional biomarkers. In a pooled analysis of two large, community-based cohorts with a median follow-up of 15.9 years, elevated circulating levels of gut microbe-derived metabolites, including TMAO and choline, were independently associated with increased risk of incident HF, even after adjustment for traditional cardiovascular risk factors [47]. In a recent meta-analysis, Li et al. [48] reported that elevated circulating TMAO levels were significantly associated with increased risks of all-cause and cardiovascular mortality among patients with HF, reinforcing its prognostic relevance as a gut-derived metabolite implicated in disease progression. These findings underscore TMAO as a mechanistically active and prognostically potent gut-derived metabolite, linking dietary precursors and microbial metabolism to adverse cardiac remodeling, systemic inflammation, and long-term mortality in both stable and acute HF contexts.

SCFAs, primarily acetate, propionate, and butyrate, are generated through microbial fermentation of dietary fibers by commensal taxa such as Faecalibacterium prausnitzii, Roseburia, and Eubacterium [44]. Recent evidence indicates that reduced fecal concentrations of butyrate are linked to heightened systemic inflammation and adverse cardiovascular outcomes, underscoring the mechanistic importance of SCFAs in maintaining metabolic, endothelial, and inflammatory homeostasis within the gut–heart axis [44]. A high-fiber diet combined with acetate supplementation was shown to protect against HF in a hypertensive model by modulating the gut microbiota, enhancing acetate-producing taxa, and reducing cardiac hypertrophy, fibrosis, and dysfunction [49]. Zuo et al. [50] showed that gut-derived SCFAs can help protect the heart by reducing inflammation, ultimately reducing atrial stress and lowering the risk of arrhythmia, which is a common and clinically significant complication in HF. Although mechanistic links remain under investigation, SCFAs are increasingly recognized as modulators of HF pathology through metabolic and immunologic pathways; further studies are needed to clarify their clinical relevance and therapeutic potential [51].

Bile acids, traditionally recognized for their role in lipid digestion, are now understood as pivotal signaling molecules linking the gut, liver, and cardiovascular system [52]. Synthesized from cholesterol in the liver and subsequently transformed by intestinal microbiota into secondary bile acids, they regulate diverse metabolic and inflammatory processes [53]. Clinical evidence linking bile acid metabolism to HF remains inconclusive, as studies show only modest associations between altered bile acid profiles and cardiac outcomes, underscoring the need for larger, stratified investigations integrating gut microbiome, dietary, and pharmacologic factors [16]. Mayerhofer et al. [54] reported that patients with chronic HF exhibit an increased secondary-to-primary bile acid ratio, reflecting microbiota-driven alterations in bile acid metabolism. However, its association with mortality lost significance after adjustment for clinical covariates [54]. Pathological elevations in circulating bile acids impair cardiac mitochondrial metabolism and suppress fatty acid oxidation via downregulation of PGC-1α, leading to structural remodeling, bradycardia, and reduced cardiac output [55]. Jointly, the data implicate that bile acid dysregulation may contribute to cardiac metabolic stress and remodeling, but its role in HF remains incompletely defined.

LPS, a structural component of the outer membrane of Gram-negative bacteria, has emerged as a pivotal pro-inflammatory mediator within the gut–heart axis [56]. Under physiological conditions, the intestinal barrier restricts translocation of LPS into systemic circulation [56]. In HF, intestinal hypoperfusion, venous congestion, and microbial dysbiosis compromise mucosal integrity, facilitating endotoxin leakage into the bloodstream. Similarly to TMAO, systemic LPS binds to TLR4 on immune, endothelial, and myocardial cells, activating downstream NF-κB and MAPK signaling cascades that amplify the release of pro-inflammatory cytokines such as TNF-α, IL-1β, and IL-6 [57]. Serum LPS of gut origin was found to correlate with elevated cardiovascular risk in atrial fibrillation, implicating microbial endotoxin translocation as a mechanistic driver of vascular dysfunction [58]. Yoshida et al. [59] found that supplementation with Bacteroides vulgatus and Bacteroides dorei suppressed intestinal LPS biosynthesis, reduced systemic endotoxin burden, and attenuated vascular NF-κB–mediated inflammatory signaling. These effects mitigated inflammation-induced vascular remodeling and endothelial dysfunction. In severe HF reduced gut microbial diversity and elevated circulating LPS correlate with increased systemic inflammation, oxidative stress, and endothelial dysfunction [60]. These findings highlight gut-derived LPS as a driver of inflammation and vascular dysfunction in HF, suggesting the microbiome as a potential therapeutic target (Table 2).

Indoxyl sulfate, a protein-bound uremic toxin derived from gut microbial metabolism of dietary tryptophan, has emerged as a key mediator in the gut–heart axis of HF [61]. Gut microbes such as Clostridium and Bacteroides metabolize dietary tryptophan into indole via tryptophanase activity; subsequent hepatic sulfation generates indoxyl sulfate, whose circulating levels rise in HF owing to renal dysfunction and increased intestinal permeability that facilitate toxin retention and translocation [61,62]. In a pioneering effort, Lekawanvijit et al. [63] demonstrated that indoxyl sulfate exerts pro-fibrotic, pro-hypertrophic, and pro-inflammatory effects on cardiac fibroblasts and myocytes. Later, Cao et al. [64] reported that in a prospective cohort of hemodialysis patients, higher plasma indoxyl sulfate concentrations were independently associated with an increased incidence of first HF events (HR 3.49, 95% CI 1.97–6.20) over a 4-year follow-up, underscoring the prognostic significance of indoxyl sulfate as a cardiovascular risk factor even in advanced renal dysfunction. In another long-term study, Imazu et al. [65] demonstrated that elevated plasma indoxyl sulfate levels independently predicted cardiovascular death and HF rehospitalization over a 5-year follow-up, with particularly strong associations observed in patients with hypertrophic cardiomyopathy, linking indoxyl sulfate burden to adverse cardiac remodeling and elevated BNP. In a recent study, Zhang et al. [61] provided mechanistic evidence that gut-derived indole compounds exacerbate cardiac remodeling and functional decline in models of chronic kidney disease–associated HF, thereby reinforcing indoxyl sulfate as a pathogenic mediator in the gut–heart axis. Together, these converging lines of mechanistic and clinical evidence firmly establish indoxyl sulfate as both a biomarker and a pathogenic effector in HF (Table 3).

## 4. Gut–Brain Axis

The gut–brain axis is recognized as a complex, bidirectional communication system connecting the enteric and central nervous systems through multiple pathways, including neurologic, endocrine, humoral/metabolic, and immune mechanisms [66,67]. Accumulating clinical, epidemiological, and experimental evidence demonstrates that the gut microbiota substantially modulates this axis, with profound effects on mood, cognition, and overall mental health [67]. Recent evidence from systematic reviews and observational studies demonstrates that age-associated alterations in gut microbiota composition play a contributory role in the pathogenesis of cognitive Impairment and dementia syndromes, such as Alzheimer’s disease (AD) and vascular dementia [67,68,69,70,71]. Throughout aging, there is a characteristic decline in beneficial taxa, including Bifidobacteria and multiple Firmicutes subgroups, along with a parallel increase in Proteobacteria and other facultatively anaerobic bacteria such as Escherichia coli and Staphylococcus [21,72]. These microbial shifts are linked mechanistically to disruptions in blood–brain barrier integrity, increased gut permeability, heightened systemic and neuroinflammation, and abnormal production of neuroactive metabolites and amyloid proteins [73].

Notably, dysbiosis-induced changes in the microbial and metabolic environment have been observed to correlate with alterations in brain structure, neurotransmitter levels, and cognitive functions in both preclinical animal models and human cohorts with cognitive disorders [20,21]. Recent systematic reviews on healthy aging populations further suggest that gut microbiota signatures may serve as early biomarkers for Alzheimer’s disease (AD) or related dementias, even preceding the onset of mild cognitive impairment (MCI) [21,68,70,71]. Taken together, these findings highlight the gut–brain axis as a key mediator linking age-related shifts in microbiome composition to cognitive decline, underscoring the potential of microbiome-targeted interventions in the preservation of cognitive health across the aging spectrum.

### 4.1. Microbiomes

The gut microbiome plays a critical role in cognitive health, influencing brain function, behavior, and neuroimmune regulation through the complex gut–brain axis [74]. Dysbiosis, or imbalance in gut microbial communities, is strongly associated with cognitive decline, including neurodegenerative diseases such as AD, partly through modulating immune responses, neuroinflammation, and production of neuroactive metabolites like SCFAs [20,75]. These processes contribute to alterations in neural activity and cognitive functions, making the microbiome a promising target for early intervention and therapeutic strategies in cognitive impairment [75].

In a Chinese cohort study [76], AD patients exhibited reduced fecal microbial diversity and a composition distinct from amnestic MCI and healthy controls, marked by depletion of SCFA-producing Firmicutes and enrichment of pro-inflammatory Proteobacteria such as Enterobacteriaceae. Vogt et al. [77] identified significant alterations in gut microbiota composition in AD patients, including reduced abundance of Firmicutes and Bifidobacterium alongside increased Bacteroidetes and Proteobacteria, suggesting that microbial dysbiosis may contribute to AD pathogenesis via immune and metabolic disruption. To support these findings, Zhuang et al. [78] reported that AD patients exhibited significant gut microbiota alterations, including reduced abundance of Firmicutes and Bifidobacterium and increased levels of Bacteroidetes and Proteobacteria. In another study, Saji et al. [79] reported that patients with MCI had a significantly higher prevalence of Bacteroides, which was independently associated with reduced global cognitive function and increased markers of cerebral small vessel disease, including white matter hyperintensity and hippocampal atrophy. In a recent systematic review and meta-analysis of 60 studies, Jimenez-García et al. [21] found that gut dysbiosis in AD is consistently marked by elevated Bacteroides and reduced Firmicutes and Bifidobacterium, with probiotic and natural interventions showing potential to modulate these microbial shifts and improve cognitive outcomes in preclinical models.

Notably, similar microbiome patterns are linked to cognition more broadly, not just in AD, as reductions in SCFA-producing taxa and enrichment of pro-inflammatory microbes. In cognitively impaired elders, amyloid-positive individuals showed increased pro-inflammatory Escherichia/Shigella and reduced anti-inflammatory Eubacterium rectale. These taxa correlated with higher peripheral IL-1β, NLRP3, and lower IL-10, linking gut dysbiosis to brain amyloidosis–related inflammation [80]. In a systematic review of cognitively healthy older adults, Kossowska et al. [71] reported that age-related shifts in gut microbiota, characterized by reduced Bifidobacterium and Lactobacillus and increased Enterobacteriaceae and Clostridia, were associated with cognitive performance, suggesting that such microbial patterns may serve as early biological markers for AD prior to clinical impairment. Li et al. [81] demonstrated that patients with MCI exhibit gut microbiota alterations nearly identical to those observed in AD, including increased Escherichia coli abundance and reduced microbial diversity, suggesting that dysbiosis may precede clinical dementia and contribute to early amyloid pathogenesis via neuroinflammatory and barrier-disruptive mechanisms. In a systematic review and meta-analysis of 17 studies, Jemimah et al. [82] reported that gut dysbiosis in AD and MCI is characterized by reduced species richness and region-specific shifts in Bacteroides abundance, with early increases in Phascolarctobacterium during the prodromal stage, supporting the potential of microbiome signatures as early biomarkers for cognitive decline. In a fecal sample from 119 individuals with MCI and 320 cognitively normal controls, Fan et al. [83] identified 59 microbial species associated with MCI and AD pathology, including Akkermansia muciniphila and Bacteroides eggerthii, whose abundance correlated with amyloid burden, hippocampal volume, and cognitive performance, highlighting species-level microbial signatures as potential modulators of neurodegeneration. Gut microbial dysbiosis not only correlates with cognitive decline but causally contributes to neurodegeneration through SCFA-mediated microglial modulation and transmissible inflammatory signaling [84,85]. In the SILCODE study [69], Sheng et al. found that individuals with subjective cognitive decline exhibited a significant reduction in anti-inflammatory taxa such as Faecalibacterium, with a progressive decline in Firmicutes, Clostridia, and Ruminococcaceae from healthy controls to cognitively impaired participants. Collectively, these findings emphasize the pivotal role of gut microbiota in the continuum of cognitive decline, from subjective complaints to Alzheimer’s pathology, highlighting microbial dysbiosis not only as a correlate but a potential driver of neurodegeneration, and reinforcing its promise as a biomarker and therapeutic target for early intervention in cognitive disorders [20] (Table 1).

### 4.2. Metabolites

The gut microbiota acts as a dynamic metabolic organ, producing a wide array of bioactive metabolites that influence brain function and cognitive health via the gut–brain axis [86]. These metabolites are sensed by enteroendocrine and immune cells, traverse or modulate epithelial and blood–brain barrier function, and act on endothelial, glial, and neuronal targets [86].

TMAO forms in the liver engages the gut–brain axis by acting on the neurovascular unit [43,87]. Experimental data indicate that TMAO can traverse or modulate the blood–brain barrier, altering endothelial tight-junction dynamics, nitric oxide bioavailability, and oxidative stress [87,88,89]. Through the same NF-κB and NLRP3 pathways described in Section 3.2, TMAO promotes neuroinflammatory damage in the central nervous system, specifically inducing astrocyte activation, microglial priming, and neuronal senescence. In brain tissue, these effects manifest as blood–brain barrier disruption, impaired synaptic plasticity, and accelerated cognitive decline [90]. In a meta-analysis by Ren et al., [91] patients with cognitive dysfunction exhibited significantly elevated levels of TMAO. Experimental evidence indicates that elevated levels of TMAO accelerate brain aging and impair cognitive function in mice, primarily by inducing neuronal senescence and disrupting synaptic plasticity [92]. In a seminal study, Brunt et al. [87] reported that elevated TMAO concentrations across humans, mice, and cultured astrocytes were consistently associated with neuroinflammation, astrocyte activation, and impaired memory performance. In a meta-analysis of over 82,000 participants, Long et al. [93] demonstrated that elevated circulating TMAO levels were significantly associated with increased risk of cognitive impairment. Taken together, TMAO has become a key microbial metabolite implicated in the pathogenesis of cognitive decline, exerting its effects through neuroinflammatory signaling, mitochondrial disruption, and impaired protein clearance [94].

SCFAs, particularly butyrate and propionate, have been shown to influence cognitive and affective processes via multiple gut–brain signaling pathways, including direct humoral transport across the blood–brain barrier, modulation of neuroinflammation through histone deacetylase inhibition, and activation of G protein-coupled receptors expressed on neural and immune cells [95,96]. In a clinical study comparing patients with MCI and cognitively normal controls, reduced fecal levels of acetic acid and butyric acid were significantly associated with increased Aβ deposition in cognition-related brain regions, suggesting that SCFA depletion may contribute to early neurodegenerative changes [97]. In a cross-sectional study of cognitively impaired individuals, distinct plasma SCFA profiles, marked by elevated acetate and valerate and reduced butyrate, were associated with AD pathology and neurodegeneration-related biomarkers, suggesting that SCFA imbalance contributes to cognitive decline [98]. In a mechanistic study, microbiota-derived SCFAs were found to attenuate key neuropathological processes in AD by interfering with Aβ peptide aggregation, complementing their known roles in modulating neuroinflammation, epigenetic regulation, and energy metabolism [99]. Fecal SCFA levels progressively decline across cognitive stages, with lower concentrations linked to poorer MMSE and MoCA scores and greater disease severity [100]. Taken together, the evidence supports SCFAs, particularly butyrate and propionate, as actionable targets to stabilize gut–brain signaling and slow cognitive decline.

Bile acids, traditionally recognized for their role in cholesterol clearance and lipid digestion, have emerged as key signaling molecules within the gut–brain axis [101,102]. As demonstrated by Nho et al. [101], elevated serum ratios of gut-derived bile acids were significantly associated with lower cerebrospinal fluid Aβ levels, increased tau pathology, and widespread cortical atrophy. Elevated serum ratios of bacterially derived secondary to primary bile acids were significantly associated with worse cognitive performance and increased risk of progression from MCI to AD [102]. Using targeted serum metabolomics and longitudinal neuroimaging, Varma et al. [103] demonstrated that lower concentrations of primary bile acids, were significantly associated with increased brain amyloid deposition, accelerated white matter lesion accumulation, and faster brain atrophy, particularly in males. Given the shared interactions between bile acids and gut microbiota, and their capacity to modulate neural, immune, and endocrine pathways, disturbances in bile acid signaling may serve as both a marker and mediator of gut–brain axis dysfunction in AD [104] (Table 2).

LPS, a potent endotoxin derived from Gram-negative bacterial cell walls, is widely used to model systemic inflammation and neuroimmune activation [105]. Its relevance to AD and cognitive decline stems from its ability to trigger microglial activation, cytokine release, and neuronal injury, especially in the hippocampus [106]. Elevated LPS levels have been shown to promote amyloid beta and tau aggregation, activate microglia, and drive synaptic loss in AD [107]. In an animal study, Zhao et al. demonstrated that both intraperitoneal and intracerebroventricular administration of LPS in mice led to dose-dependent cognitive impairment, hippocampal neuronal loss, and microglial activation, accompanied by elevated levels of TNF-α, IL-1β, and nitric oxide, as well as increased amyloid beta accumulation [108]. In humans, LPS exposure induces C-reactive protein (CRP) expression [109,110,111] and a study by Walker et al. [110] reported that elevated midlife inflammatory markers, including CRP and fibrinogen, were associated with significantly steeper 20-year cognitive decline, especially in memory domains. Completing these findings, Kaplin et al. [112] found that IL-6 release from LPS-stimulated peripheral blood mononuclear cells was significantly correlated with worse verbal fluency and higher neuropsychiatric symptom scores in AD patients. While these findings implicate LPS in neuroinflammation and cognitive decline, further research is needed to clarify its causal role in dementia and AD progression (Table 4).

## 5. Discussion

The intersection of population aging, HF, and cognitive decline is reshaping cardiovascular care and public health. In the United States, the absolute number of adults living with HF is climbing and the HF population is aging, with a mean age at hospitalization in the early 70s and projections exceeding 11 million cases by 2050 [1,2,3]. Because survival after diagnosis has improved, a growing proportion of patients are living long enough to experience the neurocognitive sequelae that undermine independence and self-management, contributing to increased hospitalizations and long-term care needs [2,5]. This demographic shift is reflected in projections showing HF-related costs exceeding $70 billion annually, with inpatient care accounting for the majority of direct expenditures [2,113]. Cognitive Impairment is frequent across the HF spectrum, associated with worse performance in executive function, attention, memory, and language, and portends rehospitalization and mortality, consequences that scale to the societal level through increased caregiving needs and health expenditures [4,5,6,7,8]. Framing cognitive Impairment as a predictable companion of chronic, late–life HF rather than a rare complication justifies routine cognitive phenotyping for interventions that address neurocognitive vulnerability alongside hemodynamics and congestion [114,115].

Beyond epidemiology, converging mechanistic evidence places the gut at the center of a shared biology linking HF and cognition [14,15]. Hemodynamic changes in HF, low forward flow and venous congestion, compromise intestinal perfusion, promote mucosal edema, and disrupt the gut vascular barrier, promoting dysbiosis and translocation of microbe associated molecular patterns such as LPS [23]. Downstream activation of TLR4/NF-κB and allied pathways fuels endothelial dysfunction and systemic inflammation that accelerate adverse cardiac remodeling [116], while the same inflammatory programs amplify microglial activation, compromise the blood–brain barrier, and injure the neurovascular unit implicated in cognitive decline [117]. Alongside, low cardiac index, vascular stiffness, and chronic inflammation impair cerebral perfusion and injure the neurovascular unit, which can predispose to cognitive impairment and dementia [118]. In return, neuroinflammation and neurovascular damage can alter the CNS activation, which has a direct effect on cardiovascular system and HF pathophysiology [119]. Moreover, HF is marked by a metabolite signature, elevated microbially derived TMAO, depletion of SCFA producing taxa, altered bile acid signaling, and accumulation of indoxyl sulfate in the setting of renal dysfunction [16,120]. Complementary observations in cognition include higher circulating TMAO, reduced fecal SCFAs, and bile acid patterns linked to amyloid/tau pathology and cortical atrophy [121]. Across HF and cognitive phenotypes, gut microbial communities exhibit reduced alpha diversity, marked by depletion of anti-inflammatory taxa and enrichment of pro-inflammatory and fermentative lineages like Escherichia, Shigella, and Klebsiella [122,123].

Rather than viewing dysbiosis and barrier dysfunction as secondary consequences of advanced disease, this vision suggests that microbiome alterations may precede, drive, or amplify both cardiac and cognitive pathology at an early stage [19,124]. The concept of the microbiome as a metabolic organ offers unique opportunities for early biomarker discovery, risk stratification, and therapeutic intervention. With advances in automated sequencing, metabolomics, and functional microbiome analysis, it is now feasible to identify microbial and metabolite profiles that not only can predict the disease onset profile of HF and cognitive impairment but also serve as modifiable targets for precision prevention. A translational approach grounded in this axis shifts emphasis from symptom management to anticipatory, upstream intervention. Modulating the gut microbiome through diet, targeted probiotics, prebiotics, precision nutrition, or more advanced technologies such as engineered biotherapeutics or microbiome transplantation appears as a promising frontier for HF and cognitive decline [72,120,125,126]. Moreover, gut-derived biomarkers such as TMAO, SCFAs, and LPS could help guide timing, intensity, and monitoring of such therapies to optimize outcomes. Clinically, HF services can incorporate routine cognitive screening and basic gut-axis risk phenotyping, dietary quality, constipation, congestion profile, recognizing their tractability and impact on outcomes. A bidirectional lens also acknowledges the role of brain and cardiac signals in shaping gut physiology, reinforcing the need for holistic approaches that integrate cardiovascular, neurological, and gastroenterological care teams.

Trials are needed to establish causality between gut-axis interventions and outcomes in HF patients with cognitive vulnerability. Despite promising associations between gut microbiota and cardiovascular outcomes, most available data are derived from cross-sectional studies, limiting causal inference. Additionally, heterogeneity in sequencing platforms, sample processing, and taxonomic resolution complicates direct comparisons across studies and may introduce bias. These challenges are further compounded by the typically small sample sizes of microbiome investigations and the difficulty of adequately controlling for major confounders, including dietary patterns, age, medication use, and comorbid conditions. These methodological limitations highlight the need for longitudinal and interventional studies to establish causal relationships and mechanistic precision. Longitudinal HF cohorts could combine serial gut metagenomics and plasma metabolomics with structural and functional brain MRI and standardized neurocognitive testing, while interventional studies assess whether shifts in microbial and metabolomic profiles precede or parallel changes in HF status, brain imaging markers, and cognitive performance. Future trials incorporating multi-omics, neuroimaging, and cognitive endpoints will be critical to define the temporal and directional dynamics of the gut–heart–brain axis in HF population.

## 6. Conclusions

The gut–heart–brain axis represents a key integrative framework linking HF, systemic inflammation, and cognitive impairment through gut microbiome dysbiosis and metabolite alterations. This axis offers promising avenues for early biomarker discovery and precision interventions targeting microbial populations and metabolites like TMAO, SCFAs, and LPS. Future clinical strategies should prioritize routine cognitive and gut-axis risk assessments in HF management. However, causal relationships and therapeutic efficacy require validation in prospective, multi-omics, and interventional clinical trials, including the design of microbiome-targeted interventions that evaluate both cardiovascular and neurocognitive outcomes. Overall, modulating the gut microbiome holds significant potential to improve cardiovascular and neurocognitive outcomes in HF patients.

## Figures and Tables

**Table 1:** Comparative Gut Microbiome Signatures in Heart Failure and Cognitive Impairment.

Feature	Heart Failure	Cognitive Impairment
**Overall** **Diversity**	↓α-diversity in HFpEF/HFrEF	↓α-diversity in AD/MCI
**Dominant** **Phyla**	↓Firmicutes; Proteobacteria, Actinobacteria	↓Firmicutes, Bifidobacterium, Bacteroidetes & Proteobacteria
**Pathobiont** **Enrichment**	↑Enterococcus, Streptococcus, Escherichia/Shigella, Klebsiella	↑Escherichia/Shigella, Bacteroides
**Loss of** **Beneficial Taxa**	↓SCFA producers: Faecalibacterium, Eubacterium, Ruminococcaceae	↓SCFA producers: Eubacterium, Bifidobacterium
**Functional** **Implication**	barrier dysfunction, cardiac inflammation	Blood–brain barrier disruption, microglial activation, cognitive decline

**Table 2:** Gut Microbiota–Derived Metabolites and Their Mechanistic Associations with Heart Failure and Cognitive Impairment.

Metabolite	Heart Failure	Cognitive Impairment
**TMAO**	Elevated; Promotes myocardial fibrosis, adverse remodeling, and endothelial dysfunction.	Elevated; Crosses the blood–brain barrier, drives glial activation and neuroinflammation.
**SCFAs**	Depleted; Weakens gut barrier, increases inflammation, and favors hypertensive cardiac remodeling.	Reduced; imbalances correlate with amyloid deposition and neurodegeneration biomarkers.
**Bile acids**	Altered profiles; mechanistic links to cardiac metabolic stress.	Altered profiles; higher gut-derived bile acids ratios associate with Amyloid-beta, cortical atrophy, and worse cognition.
**LPS**	Translocation from leaky gut; associates with systemic inflammation, endothelial activation, and myocardial dysfunction.	Potent driver of neuroinflammation; promotes amyloid/tau pathology and cognitive deficits in models.
**Indoxyl Sulfate**	Elevated; Associates with cardiac fibrosis, hypertrophy, and adverse ventricular remodeling.	-

**Table 3:** Gut-derived Metabolites in Heart Failure: Key Studies, Sample Sizes, and Translational Implications.

Metabolite	Study	Design	Sample Size	Translational Implication
TMAO	Tang et al., 2014 [45]	Observational	720	Prognostic biomarker; target for dietary/microbiome modulation in chronic HF.
	Suzuki et al., 2016 [46]	Observational	972	Improves risk stratification in acute HF
	Tang et al., 2024 [47]	Observational	11,768	Early risk marker for HF; supports primordial prevention interventions.
	Li et al., 2022 [48]	Meta-analysis	13,425	Robust prognostic biomarker; justifies TMAO-lowering strategies.
SCFA	Marques et al., 2017 [49]	Preclinical	–	SCFA supplementation may prevent/attenuate hypertensive heart disease and HF.
	Zuo et al., 2022 [50]	Preclinical	–	SCFA restoration is a therapeutic adjunct in AF and HF with AF.
Bile acids	Mayerhofer et al., 2017 [54]	Observational	162	BA profiles may help identify high-risk HF patients
	Desai et al., 2017 [55]	Preclinical	–	BA dysregulation directly contributes to HF; potential therapeutic targets.
LPS	Pastori et al., 2017 [58]	Observational	912	Circulating marker of gut–heart inflammation; target for barrier repair to prevent HF events.
Indoxyl sulfate	Cao et al., 2015 [64]	Observational	258	Risk biomarker for HF in ESRD; target for interventions.
	Imazu et al., 2020 [65]	Observational	165	Prognostic biomarker beyond advanced CKD; mediates cardiac remodeling.
	Lekawanvijit et al., 2010 [63]	Preclinical	–	Lowering indoxyl sulfate might be a strategy to slow HF progression (especially in CKD–HF overlap).

AF; Atrial Fibriation, BA; Bile Acids, ESRD; End Stage Renal Disease, CKD; Chronic Kidney Disease

**Table 4:** Gut-derived metabolites and cognition: key studies, sample sizes, and translational implications.

Metabolite	Study	Design	Sample Size	Translational Implication
TMAO	Ren et al., 2025 [91]	Meta-analysis	1675	Risk biomarker and modifiable target to slow neurodegeneration
	Li et al., 2018 [92]	Preclinical	-	Supports TMAO-lowering strategies for dementia prevention
	Brunt et al., 2021 [87]	Mixed translational study	-	Active mediator of glial activation and cognitive decline
	Long et al., 2024 [93]	Meta-analysis	≈82,000	Use in risk stratification and monitoring prevention trials
SCFAs	Gao et al., 2023 [97]	Cross-sectional	82	Non-invasive biomarkers for early AD detection (fecal SCFA depletion)
	Marizzoni et al., 2025 [98]	Cross-sectional	85	Risk stratification reflecting amyloid/tau-independent neurodegeneration
	Ho et al., 2018 [99]	Preclinical	-	Selective SCFA modulation for anti-amyloid and neuroprotection
	Wu et al., 2021 [100]	Cross-sectional	77	Markers for monitoring microbiome-targeted therapies.
Bile acids	Nho et al., 2019 [101]	Observational	1562	Blood-based indicators of core AD pathology (A/T/N status)
	Mahmoudian Dehkordi et al., 2019 [102]	Observational	1464	Prognostic markers for MCI-to-AD conversion and therapeutic targets
	Varma et al., 2021 [103]	Observational	~26,000	Supports careful evaluation of BA-modifying drugs in dementia risk
LPS	Zhao et al., 2019 [108]	Preclinical	-	Gut-derived endotoxemia relation to dementia pathophysiology
	Kaplin et al., 2009 [112]	Observational	10	Exploratory biomarkers for central neuroinflammation

## Data Availability

No new data were created or analyzed in this study. Data sharing is not applicable.

## Abbreviations

The following abbreviations are used in this manuscript:

Aβ	Amyloid-beta
AD	Alzheimer’s Disease
BNP	B-type Natriuretic Peptide
CRP	C-Reactive Protein
CSF	Cerebrospinal Fluid
HF	Heart Failure
HFpEF	Heart Failure with Preserved Ejection Fraction
HFrEF	Heart Failure with Reduced Ejection Fraction
HR	Hazard Ratio
IL-1β	Interleukin-1 Beta
IL-6	Interleukin-6
LPS	Lipopolysaccharide
MAPK	Mitogen-Activated Protein Kinase
MCI	Mild Cognitive Impairment
MMSE	Mini-Mental State Examination
MoCA	Montreal Cognitive Assessment
NF-κB	Nuclear Factor Kappa B
NLRP3	NLR Family Pyrin Domain Containing 3 (Inflammasome)
PGC-1α	Peroxisome Proliferator–Activated Receptor Gamma Coactivator 1-Alpha
SCFA	Short-Chain Fatty Acid
TLR4	Toll-Like Receptor 4
TMAO	Trimethylamine-N-oxide
TNF-α	Tumor Necrosis Factor-Alpha
